# Progress in Medicine

**Published:** 1896-10-24

**Authors:** 


### Progress in Medicine.

### RHEUMATISM AND GOUT.

**Acute Rheumatism.**—Sir W. Wade (*B. M. J.*, April 4th) has argued in favour of the view that this is a secondary disease arising by the absorption of microbes or their products from the lacunæ of the tonsils, where these microbes have grown and multiplied. Tonsillitis, indeed, may have passed away before the rheumatism begins, or may occasionally follow it, as a relapse occurs in fevers; but more often it appears just before or separated from it by a relaxed throat. It is not, indeed, every tonsillitis that is followed by rheumatism, but only when the body is in a favourable state for absorption of the infection. The practical indication is the need of curing and disinfecting both tonsils and throats at an early stage, especially if there is any tendency to rheumatism in the patient, by lotio nigra, sulpho-carbolate of zinc, salol, and other agents. Another paper on the same subject by Goedel (*B. M. J.*, June 27th) also notices the importance of early throat treatment in preventing attacks of acute rheumatism. Two explanations of the connection between the throat and the rheumatism are given. Either the epithelium of the throat is so injured by the local inflammation that the rheumatic poison enters there, or possibly in these cases we have not to do with true rheumatism, but with pyæmic arthritis. Indeed, many of them are marked by rashes and obstinacy under salicylate treatment; and in default of a specific microbe, the great variety of clinical symptoms in rheumatism tends to show that there are many different diseases classed under this name. This has been brought out in the recent discussions on erythema exudativum and purpura. Osler and Stephen Mackenzie (*Birmingham Med. Rev.*, May) differ as to whether the former is truly rheumatism or not. Very many cases have a rheumatic history, and are closely related to it. The symptoms of an attack of erythema exudativum often run a typical course of their own. Violent abdominal pains and crises may continue for weeks; there is sometimes fever, sometimes a normal temperature, remarkable transient swelling of the joints, which is rather around than in the joints, patches of local œdema or urticaria, and purpuric rashes; sometimes hæmorrhages from the mucous membranes, and very frequently nephritis, as shown by albuminuria. There may be endocarditis, or pneumonia, as sequelæ of an attack. Purpura occurring in ordinary rheumatism again differs from true peliosis rheumatica. According to Bradford (*Clinical Jour.*, Aug. 5th) purpuras are either secondary or primary. The former include those seen in heart disease and thrombosis (which are probably toxic), that in carcinoma and renal disease and tabes, and that caused by poisons, such as potassium iodide and nitrate. Among primary forms he reckons those without and those with hæmorrhage, and peliosis. The last is unaffected by salicylates, has no cardiac complications, and often causes violent diarrhœa and intestinal hæmorrhage from the sloughing ulcers of the mucous membrane, where a sub-mucous hæmorrhage has taken place. The first symptoms are joint pains, then slight swelling round the joints with pyrexia and a rash starting from the region of the joints. This rash commences as papules, hæmorrhage takes place into their centres, and they change into ordinary petechiœ. Not only in the mucous membranes, but in the skin, may the large extravasations produce sloughing with cicatricial contractions. Thus he mentions a case where the perineum and labia were affected. In these severe forms of purpura hæmorrhage may also occur in the brain, the sheathes of the optic nerves, the retina, the peritoneum, the pleura, and the pelvis of the kidney. The outbreaks in hæmophilia, again, sometimes commence with joint pains, though at first sight nothing can be more unlike than this disease and ordinary purpura. Erythema nodosum, which seems to be closely related to the rheumatic group, stands midway between erythema and purpura in one respect. For in the latter we have petechiœ unpreceded by swellings, in pure erythema swellings without petechiœ, while in E. nodosum and peliosis we find a swelling into which hæmorrhages occur. Kaposi ("Dis. of the Skin") notices that patches of erythema may appear on other parts of the body while E. nodosum is running its regular course on the legs, and perhaps with petechiœ and pains round the joints. On the whole, while many of these hæmorrhages are produced by other causes, some types have a strong rheumatic history, and others appear in the course of typical rheumatism. The relation of chorea to rheumatism is exactly parallel. Still most cases of rheumatism show no tendency to hæmorrhages, though there is probably great destruction of the corpuscles. E. Sansom (*B. M. J.*, May 16th), discusses several cases of influenza where pyrexia, swelling, and pain in the joints closely simulated acute rheumatism. Careful examination, however, showed little or no effusion within the joints. The ends of the bones were enlarged and were the seat of pain. The patients had no history of rheumatic proclivity, and the sequelæ were rather those of diseases of the nervous system than of rheumatism. In some cases the disease appeared to be acute rheumatoid arthritis. Pericarditis and endocarditis were entirely absent in the instances quoted, though dilation of the heart and vagus affections may occur. Weiss (*B. M. J.*, May 16th), has treated ten cases of rheumatic fever with serum from convalescents. No specific cure resulted, but in some cases a fall of temperature with reduction of swelling and pain and somewhat rapid recovery took place. Pouchet's (*Med. Chron.*, June) view of the action of the salicylates is that they are decomposed in inflamed tissues by the $CO_2$, which combines with the soda. The salicylic acid acts on the protoplasm and lowers the activity of the cells, and thus abolishes the inflammation. He confesses that the view of an antiseptic action on the unknown cause of the disease is possibly true, but prefers the above explanation. Treatment in rheumatism and allied affections by local hot dry air baths has been carried out with considerable success by W. Knowsley Sibley (*Lancet*, August 29th). Rheumatoid arthritis and chronic affections at once showed great relief from pain. The temperatures employed were from 200 to 270 for nearly an hour by means of the Tallerman-Sheffield apparatus applied to the affected joint only. In some cases daily local baths were given, and great improvement in the power of movement was obtained, as well as freedom from pain. No weakening or depressing effect on the heart was noticed.

**Gout.**—As to treatment, Ottinger (*Therap. Gazette*, June 15th) relies on diluents and benzoate of sodium, local applications of salicylates, and colchicum, salicylates, or antipyrin for the pain, attention being also paid to the heart and kidneys. Murrell (*Lancet*, June 6th) uses locally a liniment formed of pot. iod. ʒss., liniment sap. ℥i, ol caryoph et ol. cajuputi. āā. ʒss, sp. methyl. ℥x. In thoroughly robust patients he gives drachm doses of colchicum wine, with ten grains of pot. iod., thrice daily. This causes sharp purging and cuts short the attack. Lauder Brunton (*Practitioner*, July and Feb., 1894), in speaking of the irritable temper seen in gout and too many other ailments, finds that great relief is given by an occasional "temper powder" of twenty grains of bicarbonate of potash and ten or twenty of bromide. Similarly in migrane and the irritable temper of heart disease, like doses of salicylate of soda and bromide have a wonderful effect. Sir Alfred Garrod (*Lancet*, May 30th) lays down that guiacum has some power of relieving acute attacks of gout, and still more of preventing them if taken regularly. Moreover, it is quite harmless, even if taken for long periods. Serpenutary is to some extent a substitute. He believes that uric acid is formed by the direct action of the kidney on the urea and other nitrogenised substances in the blood. It is not formed in the system and thrown out by the kidneys, but when it is found in the blood it has been absorbed from the kidneys themselves. Guiacum thus stimulates the kidney to excrete the uric acid. Murrell agrees as to the value of guiacum, but gives twenty grains of guiacum resin in a confection three times a day; and Haig looks on guiacum as suppressing gout by rendering the blood more acid, though it does not eliminate the poison. We may add that Garrod usually employs the powder or the ammoniated tincture. The effect of sugar and champagne in gout are frequently alluded to, but George Harley (*Lancet*, Aug. 1st) denies that sugar has any influence. Great eaters of sweets, women in Eastern harems, and negroes are not gouty; nor is sugar a nitrogenous matter like uric acid. Subjects of hereditary gout have been unable to evoke any symptoms when they have experimentally taken very large quantities. Much of the dry champagne now sold is simply an immature wine to which little or no sugar has been added. Harley thinks that this wine, from the acetic acid it contains and its newness, is far more dangerous than the sweetened kinds, especially if these are mellowed by age. Boyd (*Dublin J. Medical Sciences*) points out that treatment of one system of the body in gout, such as the digestive, or, indeed, of elimination is insufficient; and that in the gouty patient metabolism is defective everywhere. In the nervous system, in the circulatory, lymphatic, excretory, muscular, and respiratory mechanisms there is alike a failure to carry out the normal changes and oxidations. Irregular formations of leucocytes, of urea, and carbonic acid abound in every part of the gouty patient, and no single one of these is responsible for the entire disease.

**Rheumatoid Arthritis.**—A. H. Carter (*Lancet*, Aug. 29th) describes a case of localised arthritis affecting the right shoulder. The deltoid supra and infra-spinatus muscles were completely atrophied. There was no implication of other joints, except a history of pains before the course of the disease began in this shoulder. The man was a brassworker, and the question arose whether the joint lesion and the muscular wasting had to do with the primary neuritis. W. Armstrong (*B. M. J.*, May 16th), believing that R. arthritis depends on nerve irritation, classifies cases as to their origin as rheumatic, gouty, utero-ovarian, neurotic, strumous, and those from local injuries. He finds most benefit from galvanic baths and wine of cod-liver oil and tonic remedies, with a generous diet. Hydropathy may be useful or the reverse; hot baths especially are generally injurious. In the rheumatic forms hydropathy gives more promise of success, and the so-called wet massage is often valuable in all forms. The electric baths, with constant and alternating currents, especially seem to have given good results at Buxton. S. Hyde (*Lancet*, May 16th) treated 200 cases of sciatica at Buxton with the waters and tonics. He reports 45 per cent. cured, 50 per cent. improved. He finds that it occurs equally on the two sides, has little or no relation to constipation, and in only 13 per cent. was there any history of chills, so that his experience is rather strongly opposed to the common view of the causation and pathology of this painful state.

